# Whole strains vs MGEs in short and longterm transmission of ESBL genes between healthcare and community settings in Uganda

**DOI:** 10.1038/s41598-023-35879-x

**Published:** 2023-06-23

**Authors:** Yaovi Mahuton Gildas Hounmanou, Agnes Wanyana, Stephen Alafi, Fred Wabwire-Mangen, Henrik Christensen, John Elmerdahl Olsen, Denis Karuhize Byarugaba

**Affiliations:** 1 Department of Veterinary and Animal Sciences, Faculty of Health and Medical Sciences, University of Copenhagen, Frederiksberg C, Denmark; 2 College of Veterinary Medicine, Animal Resources and Biosecurity, Makerere University, P.O. Box 7062, Kampala, Uganda; 3 School of Public Health, Makerere University, P.O. Box 7062, Kampala, Uganda

**Keywords:** Next-generation sequencing, Bacterial infection, Antimicrobial resistance, Molecular biology, Bacterial genetics

## Abstract

Multidrug-resistant ESBL-producing *Escherichia coli* are a leading cause of infections in hospital and community settings. Based on samples from two hospitals in Uganda and households of inpatients we tested the hypothesis that ESBL *E. coli* and/or their resistance determinants could spread within the healthcare and community settings through discharged patients that were still colonized. We used bacterial culture, susceptibility testing whole genome sequencing and detailed bioinformatics analysis to test the above hypothesis. Genome analysis revealed presence of predominantly *bla*_CTX-M-15_ and *bla*_OXA-1_ genes with a total resistome with genes belonging to 14 different classes of antimicrobials. Short-term cases of strain sharing were reported within each setting and strains from the two settings were found to cluster together based on their overall resistome. Long-term horizontal transfer of ESBL genes by various *Inc*F and *Inc*Y types of plasmids shared between healthcare and community settings was demonstrated. Based on hybrid assembly, plasmid reconstruction and phylogenetic analyses, our study suggests that while the dissemination of AMR between healthcare and community settings in the short-term is possible at whole strain level, the long-term transmission between healthcare and communities is sustained by the transfer of plasmids circulating across niches and disseminating related resistomes.

## Introduction

The spread of AMR bacteria in humans, animals, and the environment causes increased morbidity and mortality, which is associated with a high economic burden^1^. *E. coli* is one of the main pathogens causing hospital acquired-infections in both developed and developing countries, and is also a leading cause of infections in the community and in livestock^2,3^. ESBL *E. coli* are one of the key AMR threats to human health^4^. The transmission of ESBL-producing bacteria from hospitals to the community may make community acquired infections such as urinary tract infections (UTI) increasingly more complicated to treat. One study showed that the probability of transmission of ESBL-producing bacteria in the household from an index patient to a household contact was 67%, and from a household member to another was 37%^5^. This risk could be exacerbated by imprudent antibiotic use in the community, as selective pressure from antibiotics leads to further dissemination of AMR^6^. Similarly, transmission may occur within hospital environments. A study from Vietnam showed that Hospital transmission of ESBL producing *E. coli* cause a high burden of hospital acquired infection (HAIs), mortality and healthcare costs^7,8^, and that about 30% of patients in 21 Vietnamese intensive care units (ICUs) had HAIs due to ESBL-producing *E. coli*. The studies also revealed that the ESBL colonization rates increased from 13% on admission to 89% at day 15 of hospital stay^8^. Similar findings regarding the burden of ESBL in health-care facilities have also been reported in Africa^9,10^. Such situations often lead to longer duration of hospital stay and increased risk of transmission of ESBL to family members, the environment, and the community upon discharge.

Reducing non-indicated antibiotic use in the community is important to reduce spread of ESBL (and AMR in general). It is equally important to prevent the introduction of “novel” ESBL strains into the community to prevent ESBL spread in the community. Recent studies from Kenya, England and The Netherlands reported MDR strain-sharing between humans in the community and between livestock, but failed to demonstrate close relatedness between strains from these different niches^2,11,12^.

There is an overall paucity of evidence especially in developing countries regarding the transmission of MDR ESBL producing bacteria between healthcare and community settings. In this study, we tested the hypothesis that MDR ESBL producing bacteria from an inpatient could be passed down to the community members, including to their livestock, when discharged while still colonized with ESBL strains. We further, investigated whether transmission of mobile genetic elements is the source of ESBL flow from hospitals to the community. The latter part was included as the dissemination of ESBL is mainly attributed to plasmids^13^. We used Illumina short reads and Oxford Nanopore long read sequences to characterize and compare ESBL *E. coli* and plasmids from healthcare and community settings to map transmission between and within the different niches.

## Results

### ESBL carriage

Of the 1556 samples analyzed, 81.9% (1275) yielded suspected ESBL producing bacteria. The antimicrobial susceptibility testing of the obtained isolates showed that many of the strains were resistant to critical cephalosporin and carbapenem antimicrobials such as Cefepime, Cefotaxime, Cefoxitin, Ceftazidime, Ertapenem and Imipenem in both healthcare and community settings. Isolates from community settings showed similar degree of resistance to several critically important drugs as the isolates from healthcare settings (Table 1).

**Table 1:** Resistance of ESBL *E. coli* from different sources in healthcare and community settings.

Antibiotic	Health care				Community		
	Patient (n = 82)	HCC (n = 30)	HCW (n = 7)	HCE (n = 35)	HHA (n = 34)	HHC (n = 60)	HHE (n = 20)
Ampicillin	97.1%a	94.6%a,b	96.0%a,b	89.4%b	86.2%a,b	93.3%b	76.4%a
AmoxyClav	49.0%b,c	27.0%a	40.8%a,b	56.7%c	66.7%a	27.9%b	29.7%b
Aztreonam	87.0%a	88.6%a,b	79.6%a,b	75.6%b	81.0%a,b	93.3%b	67.9%a
Cefepime	82.7%a	82.9%a,b	66.7%b,c	65.0%c	86.2%a	84.1%a	50.0%b
Cefotaxime	95.6%a	94.6%a	94.1%a	91.7%a	80.0%a	95.5%a	82.0%a
Cefoxitin	24.0%a	10.8%a	41.7%b	61.2%c	35.4%a,b	17.8%b	47.7%a
Ceftazidime	74.4%a	75.7%a,b	71.4%a,b	61.1%b	67.7%a	86.4%a	50.0%b
Ceftriaxone	94.2%a	94.6%a,b	94.1%a,b	85.6%b,c	87.7%a	90.9%a	70.9%b
Cefuroxime	94.7%a	91.9%a	96.0%a	95.6%a	93.8%a	93.3%a	90.7%a
Chloramphenicol	39.4%a	24.3%a,b	21.6%b	40.3%a	26.6%a,b	17.8%b	36.9%a
Ciprofloxacin	45.7%a	29.7%a,b	35.3%a,b	29.1%b	44.6%a	31.1%a,b	20.3%b
Ertapenem	13.9%a	5.7%a	27.5%b	46.5%c	39.1%a	15.6%b	35.5%a
Gentamicin	59.1%c	29.7%a,b	34.0%a,b	43.9%b	18.5%a	24.4%a	20.9%a
Imipenem	10.3%a	3.0%a	22.4%b	34.2%b	3.2%a	7.9%a,b	19.1%b
Nitrofurantoin	29.0%b	8.6%a	22.2%a,b	62.6%c	19.0%a	22.2%a	59.5%b
Streptomycin	19.2%c	51.4%a,b	71.4%b	49.4%a	29.2%a	37.8%a	25.3%a
Sulpha/Trimethoprim	92.8%a	89.2%a,b	80.4%b,c	73.6%c	80.0%a,b	86.7%b	64.9%a
Tetracycline	76.9%a	86.5%a	78.4%a	44.1%b	63.1%a	66.7%a	43.7%b

Tests are adjusted for all pairwise comparisons within a row of each innermost subtable using the Benjamini–Hochberg correction.

Values in the same row and subtable not sharing the same subscript (a, b or c) are significantly different at p < 0.05 in the two-sided test of equality for column proportions. Cells with no subscript are not included in the test. Tests assume equal variances.

*HCC* caretaker, *HCW* healthcare worker, *HCE* healthcare environment, *HHA* household animal, *HHC* household contact, *HHE* Household environment.

**Figure 1:**
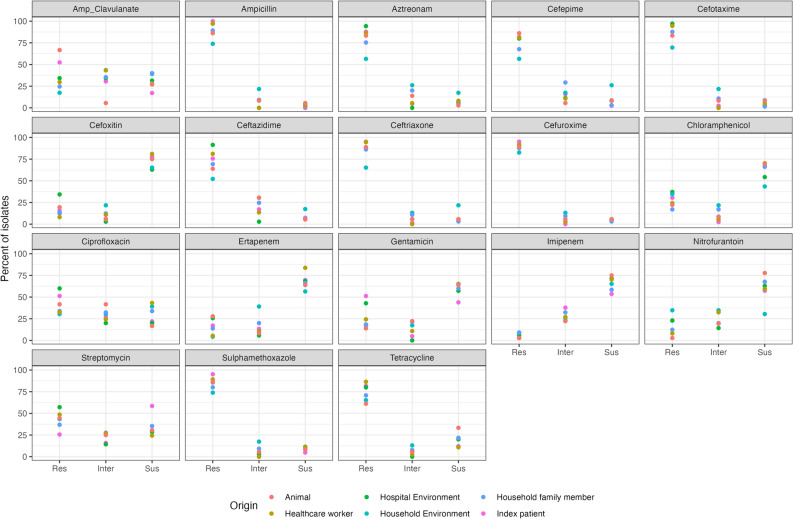
Relative frequencies of resistance, intermediate and susceptible strains by niche to each antimicrobial.

In total, 260 of the 268 ESBL *E. coli* isolates on which we performed AST were multidrug-resistant displaying resistance to at least one antimicrobial within three or more classes of antimicrobials^14^. The strains were selected based on their ESBL-status. Accordingly, isolates presented with high resistance to 4th generation cephalosporins, ranging from 65% in samples from community settings to 96% in isolates from healthcare environments (Table 1, Fig. 1).

### ESBL genes detected and resistome sharing across niches in healthcare and community settings

**Figure 2:**
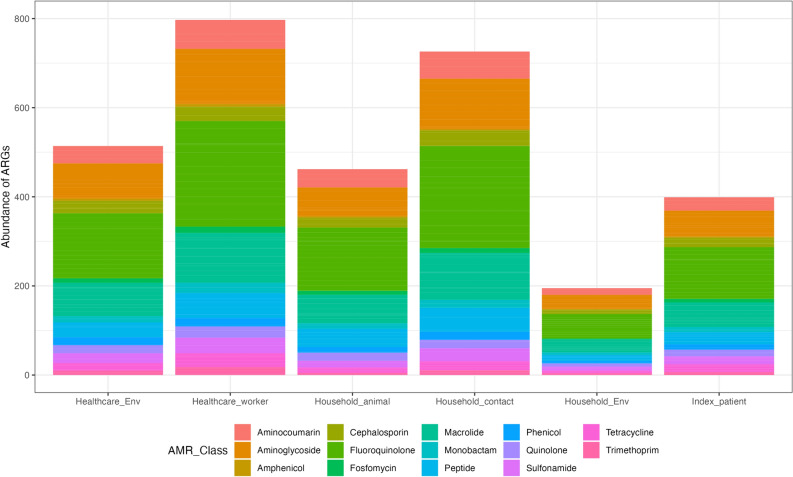
Relative abundance of antimicrobial resistance genes detected by class per niche. Although the number of ARGs vary according to the number of strains in each category, the plot shows the presence of all 14 AMR classes in all the categories.

Sixty-seven strains were sequenced, and genomes were analyzed for the presence of resistance genes. The 67 sequenced isolates were all confirmed to be *E. coli* and their overall resistome showed the presence of 14 classes of antimicrobial resistance genes in each sample source from healthcare and community settings (Fig. 2). Details on resistance genes per strain are provided in Table S1. *bla*_CTX-M-15_ was the most common ESBL gene detected (n = 41), followed by the AmpC betalactam gene *bla*_OXA-1_ present in eight samples. The least common ones were *bla*_CTX-M-55_ (n = 3), *bla*_CTX-M-3_ (n = 3), *bla*_CMY-2_ (n = 2), and then *bla*_CTX-M-25,_ *bla*_CTX-M-102,_ *bla*_CTX-M-103,_ *bla*_CTX-M-137_ (total n = 41), found in one isolate each (data shown in Table S1).

For assessing sharing of resistant strains between healthcare and community settings, an initial assessment of the AMR situation in the two compartments was performed by comparing the resistome richness based on all AMR genes detected by class of antimicrobials in the sequenced isolates. Similarity in richness would indicate similarity of strains between the two compartments and a similar selection pressure for maintenance of strains. Abundance plots showed that all niches presented indiscriminate presence of all 14 antimicrobial classes detected in their resistome, but also that some niches harbored more resistance genes than others due to the sample size (Fig. 2).

**Figure 3:**
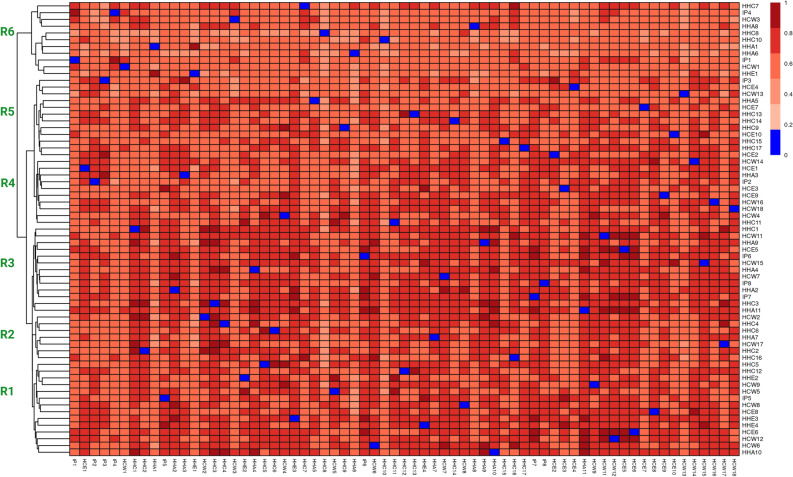
Resistome clustering based on the Jaccard similarity index between all AMR genes detected in each sample. Samples are named with the initials of the sample sources to display diversity of niches within the resistome clusters. The blue color indicates pairwise map of the same sample. *HCW* for healthcare worker and caretaker at hospital, *HCE* healthcare environment, *HHA* household animal, *HHC* household contact, *HHE* household environment.

A further analysis of the resistome revealed six shared clusters (R1–R6) based on Jaccard similarity analysis (Fig. 3). In each of the clusters, there were strains from healthcare and community settings that shared similar resistome content (Fig. 3). In the resistome cluster R1 for instance, a strain from an index patient had a closely related resistome with other strains originating from other samples from the healthcare environment as well as from the community settings including household animals, household environments and family members. This is an indication that strains in health care setting were not very different from strains in community setting with regard to resistome, as also indicated from the results in Table 1.

### Diversity of ESBL strains between healthcare and community settings

To be able to perform detailed mapping of possible transmission routes of ESBL *E. coli* from the hospital to the community, we selected strains from clusters where an index patient (admission to the hospital) was linked to ESBL isolation from health care settings and/or household members after returning home from hospital. We sequenced strains in such clusters and found that the 67 strains sequenced belonged to 42 different sequence types. Thus, the strains belonged to almost as many MLST as the number of strains sequenced in each niche, a sign of highly heterogeneous ESBL *E. coli* populations across samples from the 6 months of sampling (Table S2).

**Figure 4:**
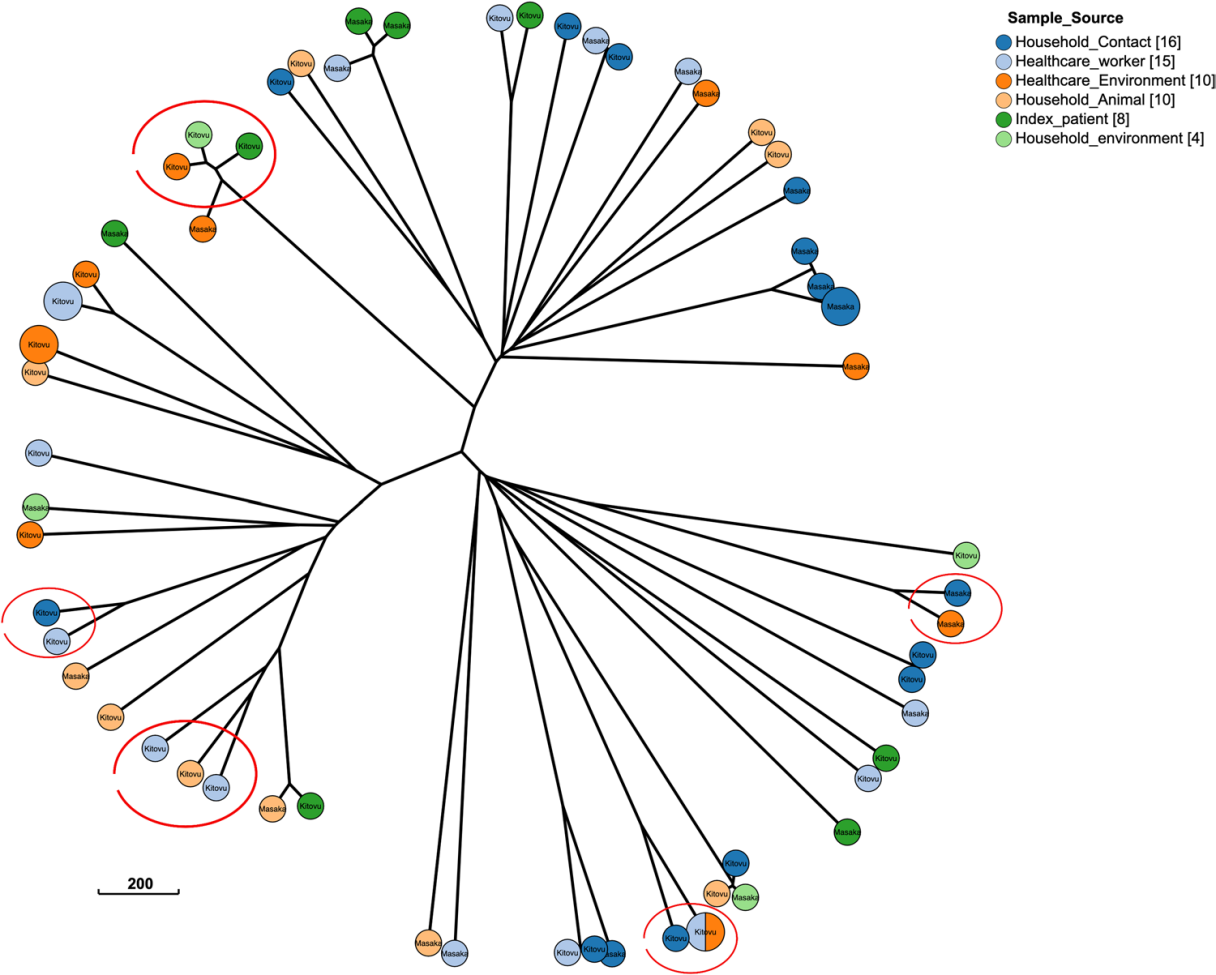
MST tree built with allelic distances generated by cgMLST from analysed strains. The red circles indicate instances where related isolates from healthcare and settings are found. Within each leave the name of the hospital is reported.

**Figure 5:**
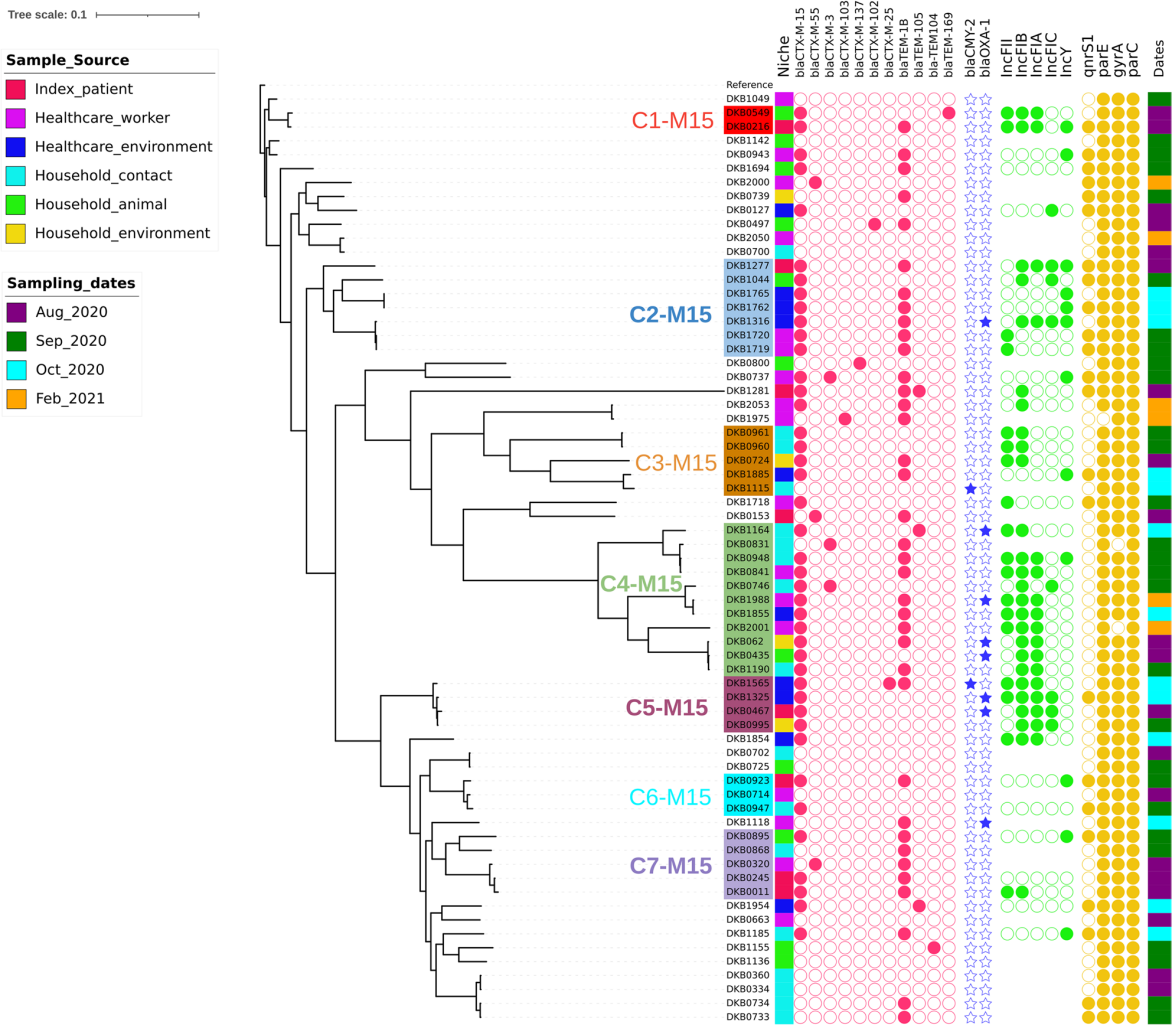
Core-genome SNP-based phylogeny of the ESBL *E. coli* strains and selected markers.

The high heterogeneity in the ESBL-*E. coli* isolates led us to the analysis of cgMLST phylogeny based on allelic distances. The results of the cgMLST analysis corroborated a wide genetic diversity between the strains isolated from each niche (Fig. 5). Based on this analysis we were able to identify up to five instances of sharing events where isolates from healthcare and community settings within strain-clusters from the same hospitals were found in the same branch and closely related, suggesting somewhat related clades of strains being transmitted between healthcare and community settings (Fig. 4).

On the other hand, core-genome SNP-based phylogeny of the strains clustered them into several unrelated lineages. With this higher resolution analysis, we identified one instance of strain transmission between healthcare and the community, and this was between the isolate DKB841 isolated from a healthcare caretaker in Kitovu hospital showing only two SNPs difference from the strain DKB948 isolated a month later at the index-patients household from another family member (clade C4-M15, Fig. 5).

A few other closely related isolates (with less than 50 SNPs apart) were identified with the SNPs analyses, but they were all isolated either within healthcare setting only or the community setting only (Fig. 5, Table S3). For instance, strains DKB0435, DKB062 and DKB1190, all from community settings (including household family member, household animal and household environment), isolated between August and September 2020 were 36–47 SNPs apart. Moreover, isolates DKB1719 vs DKB1720 as well as isolate DKB1762 vs DKB1765 isolated the same day in Kitovu hospital settings (including from environmental samples and humans) were strictly clonal with zero SNP, showing a short-term nosocomial transmission of ESBL strains within hospitals. A similar situation was observed at community level where strains DKB0960 vs DKB0961 isolated the same day showed two SNPs difference within the household of a returning patient. Similarly, strains DKB0733 and DKB0734 isolated from household contacts of a discharged patient discharged from Masaka hospital were only one SNP apart. In addition, it was observed that strains DKB0702 from a household family member was closely related (14 SNPs difference) to the strain DKB0725 isolated from a domestic animal isolated the same day related to an index patient from Kitovu hospital. So short-term strain sharing within each setting was found to be more common than across settings.

**Figure 6:**
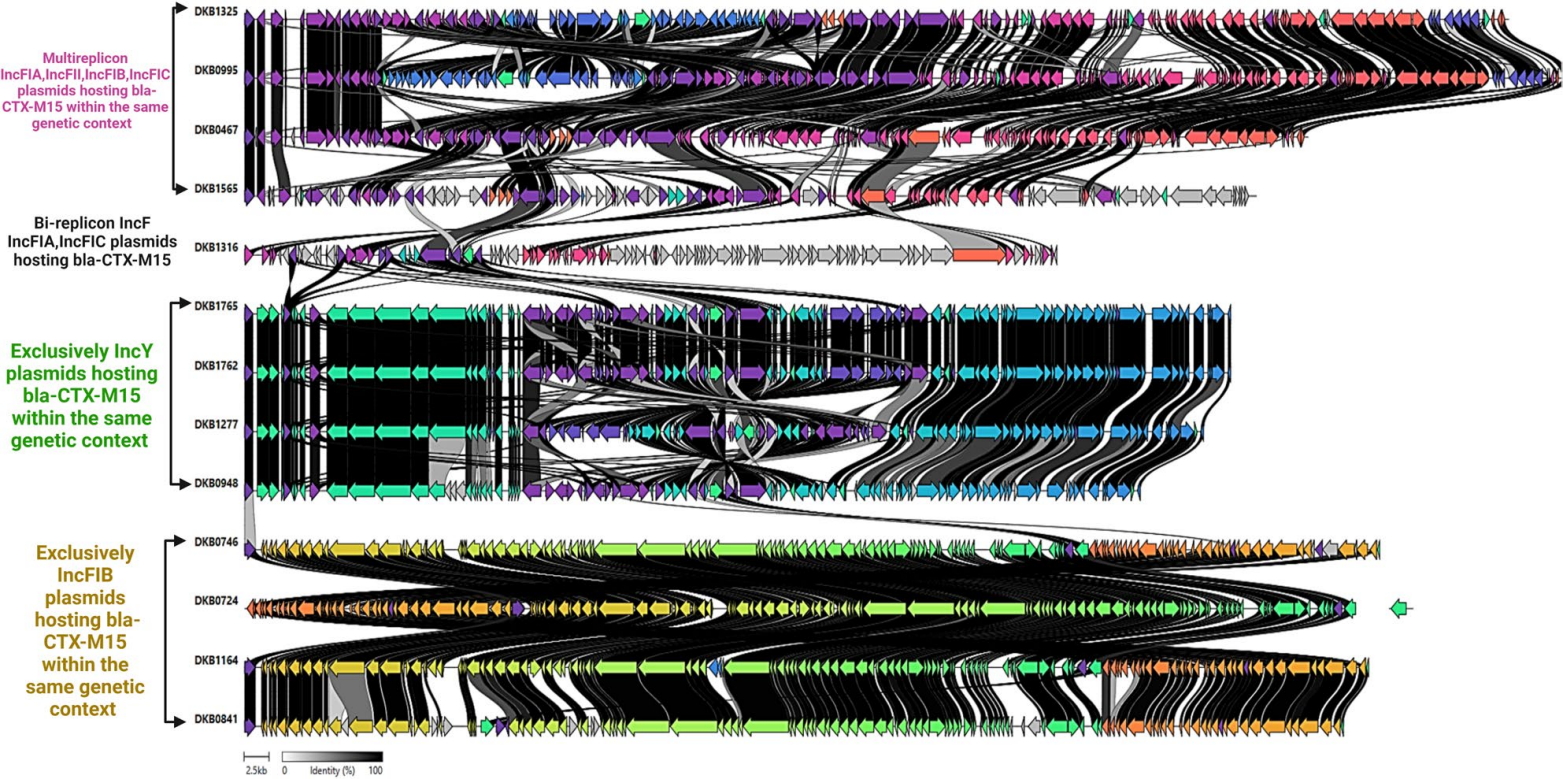
Overall comparison of plasmids categories hosting the *bla-*_CTX-M-15_ gene as reconstructed from hybrid assemblies in healthcare and community strains. The observed clustering represents the distinction between the main plasmid categories described in the study. Same genes have the same coloration and the black lines between plasmids indicate the degree of gene-by-gene similarity between and within plasmid clusters.

Overall, the SNP-analysis (Fig. 6) and the SNP distance data in Table S3 indicate that short-term transmissions of ESBL strains within community and within healthcare settings are common. However, besides the only event between the strains DKB841 and DKB0948 (2 SNPs, clade C4-M15 of Fig. 5) and the five loosely related sharing events reported by the cgMLST analysis (Fig. 4), no other cases of strain sharing across the healthcare and community settings were observed. This suggest that either the long-term continuous transmission between the two settings is mediated by another transmission mechanism, for example mobile genetic elements, or transmission is generally not followed by persistence in the new host. Based on this we investigated the role of mobile elements in the spread of ESBL genes in our study area.

### The location of ESBL genes in strains and the possible role of plasmids in disseminating ESBL genes between health care and community setting

The SNP analysis pointed to the occurrence of genetic relatedness in subclades harboring the *bla-*_CTX-M-15_ gene (Fig. 5). Isolates from different niches (in healthcare and community settings) in these clades, e.g. C1-M15 to C7-M15, harbored the main *bla-*_CTX-M-15_ gene on related types of plasmids (Fig. 5). In many cases within these clades, the *bla-*_CTX-M-15_ gene was located within a consistent genetic context on the same plasmid in samples from both healthcare and community settings (e.g., from C4-M15 and C5-M15, Fig. S1). This suggested that dissemination of ESBL genes was happening via plasmids that were circulating across strains from healthcare and community settings within these phylogenetic clades. The complete reconstruction of the plasmids from representative isolates from each clade was done using hybrid assembly of long and short-read sequences. This identified at least four categories of conjugative plasmids, based on similarities in their genetic arrangements, that were likely responsible for the long-term transmission of ESBL across settings (Fig. 6, Table S4).

As shown in Fig. 6, one of the categories of plasmids was composed of multi-replicon plasmids (IncFIA, IncFIA, IncFIB, IncFIC) of at least 100kbp nucleotides that hosted the main ESBL gene, *bla-*_CTX-M-15_, in a similar genetic arrangement (Fig. S1). Strains with this type of plasmid were detected in healthcare and community settings, suggesting a role in spread of ESBL genes between the compartments.

Another category was the *Inc*Y plasmids also carrying *bla-*_CTX-M-15_ in similar genetic contexts in the study settings. These IncY plasmids are smaller than the previous with sizes around 90 kbp and occurring in strains mainly found in the same phylogenetic clade. Our analysis also identified a group of plasmids with only the IncFIB rep type with specific genetic context also very likely responsible for the dissemination of the *bla-*_CTX-M-15_ gene across niches.

**Figure 7:**
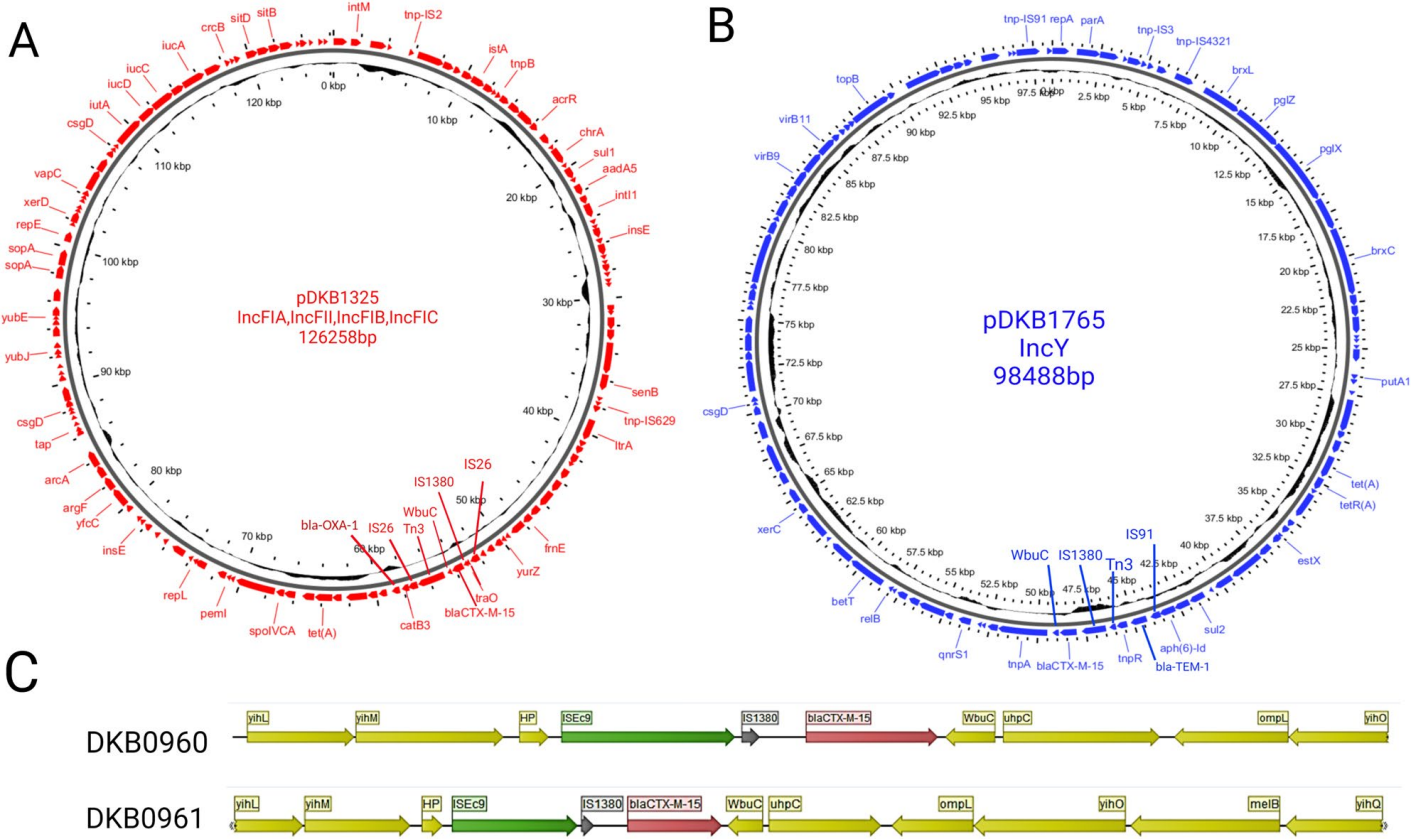
Graphical visualization of genetic features. Context of representative complete ESBL-plasmids reconstructed by hybrid assembly from strains DKB1325 (**A**) and DKB1765 (**B**). In panel (**C**), we show the genetic context around *bla-*_CTX-M-15_ in the strains where we observed that the gene was located on the chromosome.

The majority of plasmids responsible for the carriage of the *ESBL* genes across strains from healthcare and community settings were of the IncF types (Fig. 7A, Fig. S1) with the *bla-*_CTX-M-15_ gene often located upstream of a transposon insertion sequence IS1380 and varying genetic contexts downstream depending on the rep types (Fig. 7A,B).

Importantly, the hybrid assembly also allowed us to determine that in some cases like in strain DKB0960 and DKB0961, *bla-*_CTX-M-15_ was located on the chromosome within genetic contexts closely consistent to the one found on the group of *Inc*FIB plasmids (Fig. 7C). This indicates the possibility of a long-term dissemination of the IncF plasmids in the study area that lead to this settling of the ESBL gene in these isolates in the chromosome within the same context, probably through the ISEc9 and IS1380 transposases upstream of *bla-*_CTX-M-15_ in these strains.

## Discussion

We conducted a longitudinal study for 8 months following patients in the healthcare and household settings in two tertiary hospitals in Uganda. We investigated the hypothesis that multidrug resistant bacteria (with ESBL-producing *E. coli* as proxy) and/or their resistance determinants could spread within and between healthcare and community settings through discharged patients that were still colonized. After analyzing 1556 samples from different sources in the healthcare and community settings and performing different phenotypic and genotypic typing analyses, our overall findings show that the transmission of AMR between healthcare and community settings is manifold.

We confirmed a high ESBL carriage (81.9%) similar to but also different from similar studies in Uganda who reported varying prevalence from as high as 89%, 62% to below 30%^15–17^. These differences may relate to differences in sampling frameworks, varying levels of infection control in healthcare settings and detection methods but nevertheless relate to a big challenge of ESBL as a serious health threat. We report that in both healthcare and community settings, bacterial isolates displayed similar degree of resistance to several critically important drugs, indicating either sharing of strains or similar degree of selection on the bacteria in the two settings. This further suggests a common epidemiological background for strains circulating in the healthcare and community settings with the hospital being the likely source of selection of AMR based on previous evidence that one in eight patients acquired resistant bacteria upon admission to hospitals^8^. This finding is further corroborated by the genotypic resistance data where we found similarity in the resistome (resistance gene richness) of strains originating from the two compartments. It is however worth emphasizing that the sharing of resistome here is based on overall resistance gene content regardless of the overall dissimilarity between strains, and as such is not a proof of strain sharing. Nevertheless, these data overall support the hypothesis that strains from healthcare and the community settings were not very different with regard to resistome, and that they might indeed be subjected to a similar selection pressure for maintenance.

Diversity-wise, the study also showed that the strains sequenced belonged to almost as many MLS-Types as the number of strains sequenced from each source, a very common situation for the highly heterogeneous *E. coli* populations also reported in previous studies^3,13,18^. In this wide diversity, we identified up to five instances of short-term sharing events across healthcare and community using cgMLST, suggesting related clades of strains being transmitted between healthcare and community settings. This phylogenetic method which is based on cgMLST allelic distances, although “shallow”, is often used when analyzing highly heterogeneous genomes like *E coli* strains and have been useful in previous studies to attribute likely related strains^12^. Using the higher resolution phylogenetic analysis based on SNPs, we identified one instance of strain transmission between healthcare and the community, where an isolate from the caretaker of an index patient in the hospital was found closely related to an isolate obtained a month apart from a family member at the household. Altogether, these diversity analyses show that dissemination of resistant strains does occur at low level between the hospital and the community but in a short-term basis.

The study also detected several instances of short-term nosocomial transmission of ESBL strains within the hospital premises. Likewise, we report cases of very closely related strains occurring within the community indicating that short-term strain sharing within community and within healthcare settings is common. Close genetic relatedness among isolates in hospital acquired infections and within household settings has previously been documented^19^, where the authors observed that in the Swiss context, the household outweighed nosocomial transmission of ESBL, likely because of the effects of hospital infection control measures. Our study instead demonstrated similar transmission was reported in hospitals as at household levels unlike the Swiss study that is likely explained by the possible differences in the infection prevention and control differences between Swiss and Ugandan healthcare facilities. This indicates that in the Ugandan case more efforts, such as improved hygiene, may be necessary to minimize nosocomial transmission of AMR within hospitals and transmission at household levels.

The low level of long-term strains transmission between healthcare and community settings in our study suggested that prolonged transmission between the two settings is mediated by another transmission mechanism, which we found in this case was mobile genetic elements, mainly plasmids. Such mechanism of transfer is common with ESBL producers both in clinical settings^2,19^ as well as in communities^3,13,20^. The study pointed that the main ESBL gene circulating in the sequenced samples, the *bla-*_CTX-M-15_ gene, was located within a consistent genetic context on the same plasmid in samples from both healthcare and community settings for at least 6 months. It therefore suggested that it was related plasmids that were responsible for ESBL long-term transmission from healthcare and community settings. Many of the plasmids were of the conjugative *Inc*F types common in many ESBL transmission cases^3,6,13^. We also found indication that the long-term transfer of the *bla-*_CTX-M-15_ gene via these plasmids may lead to settling of the ESBL gene into the chromosome of some of the isolates, probably through the transposon insertion sequences located upstream of the gene on the *Inc*FIB plasmids. Such situations are indications of an epidemiological context, where plasmids have played the long-term mediators of horizontal transfer between the healthcare and the community severally. The role of whole strains in AMR transmission from healthcare settings to communities may require longer follow up of hospitalized patients after they return home. We did not collect follow up samples of these patients at several intervals after discharge from hospital. Despite this limitation, we demonstrated through cgMLT and resistome analysis that some patients had closely related strains and resistome from the household members, animals, and environments and the hospitals settings that support potential role of whole strains in AMR transmission between the different niches.

Overall, the data suggest a long-term well-established and continuous colonization of humans, animals, and the environment in the study area by these multidrug resistant bacteria sustained by horizontal gene transfer across niches played by different plasmids. As previously shown by other studies^2,13^ the transmission of resistance by ESBL-producing *E. coli* between different reservoirs might not be easy to assess at strains level due to the high heterogeneity of *E. coli* but can be more surely observed with plasmids and the genomic context of marker genes.

## Methods

### Sampling and sample processing

The study was conducted between July 2020 and March 2021 at and around two tertiary healthcare facilities: Masaka Regional Referral Hospital, a government public hospital, and Kitovu Hospital, a not-for Profit Faith based healthcare facility, both tertiary healthcare facilities in Uganda. Rectal swabs were collected from “index patients” admitted to the two hospitals. The inclusion criteria were that they were on antibiotic treatment and kept livestock in their homes. Rectal swabs were also taken from caretakers, cleaners, healthcare workers, and other caretakers of other patients on the same ward. These samples were grouped as “healthcare workers”. Samples from the hospital environment, including swabs of surfaces, sinks, and wastewater were taken as “healthcare environment”. When discharged, patients were followed-up to their homes, where samples were collected from their household contacts, livestock, and household environments (see details in Table S5). The sampling framework is summarized in Fig. S2. In total, we collected and screened 1556 samples from the different sources.

### Antimicrobial susceptibility testing and short-read sequencing

All samples were processed for isolation and identification of ESBL producing *E. coli* using methods as described in our previous studies^3^. *E. coli* were presumptively identified based on the standard IMViC (indole test, methyl red test, Voges-Proskauer test, and citrate utilization) tests and later confirmed upon sequence analysis. The presumptive colonies were obtained on McConkey agar containing 2 ug/ml cefotaxime, purified and confirmed as ESBL by the double-disk synergy method. Moreover, 268 of the confirmed ESBL producing isolates were subjected to antimicrobial susceptibility testing against 18 antimicrobials by disc diffusion assay (Table S5) as previously described^3^.

DNA was purified from 67 of the ESBL *E. coli* strains and subjected to short-read whole genome sequencing (WGS) on Illumia MiSeq. The selection of these 67 isolates (Table S6) was based on the criteria that the isolate belonged to sampling clusters, where ESBL isolates from index patients were present together with ESBL isolates from (either/or) their caretakers, associated family members, animals, healthcare workers, as well as hospital and community environments*.* DNA extraction, WGS and sequence reads processing was performed following previously described protocols^13,21^.

### Data processing for bioinformatics analysis

All bioinformatics analysis was performed using internally customized scripts run on the terminals of the Danish National Life science supercomputer, Computerome2 on nodes each equipped with dual 20-core Intel Xeon Gold 6230 CPUs and 192 GB RAM.

### Long read sequencing, hybrid assembly and plasmids reconstruction

After preliminary analyses of the short-read sequence data, we selected representatives of different phylogenetic clades identified in our dataset and carried out long-read whole-genome sequencing of these isolates (n = 15) using the Oxford Nanopore technology to obtain completely assembled genomes for comprehensive analyses. Genomic DNA was re-extracted from the selected isolates using the A&A Genomic Mini AX Bacteria+ kit (A&A Biotechnology, Gdańsk, Poland) to obtain less fragmented DNA. Long-read sequencing libraries were then generated using the Rapid Barcoding Sequencing Kit (SQK-RBK004) and sequenced on a MinION Flow Cell (R9.4.1) with MinKNOW version 22.08.9 (Oxford Nanopore Technologies, Inc., Oxford, United Kingdom). The super-accuracy (r9.4.1_450bps_sup) configuration was employed to basecall the Fast5 files generated using the nanopore-supported software Guppy v. 6.3.8. The basecalled FASTQ files were then concatenated into a single file per basecalled sequence run and demultiplexed into individual FASTQ files.

Before, hybrid assembly, we first filtered out poor-quality reads using fastp v0.20.1^22^ (for short reads) and Filtlong v0.2.1^23^ (for long reads) at a sequence similarity threshold of 95%, retaining only the reads with a minimum length of 1 kbp and excluding the worst 5% of the reads. Long-read sequences assembly was first performed using Tricycler ﻿﻿pipeline^24^, and the generated circularized assemblies were then polished using Medaka v1.7.2. To generate high-quality complete assemblies, the generated long-read assemblies were then polished with the short reads using Polypolish^25^. For comparison of hybrid assemblies, Unicycler v0.4.9b^23^ was then used on the filtered short and long read sequences. From the hybrid assemblies, the *mob-recon* command from the mob-suite v3.1.0^26^ software was used to separate complete chromosomal sequences from mobile elements including typing for plasmids and associated insertion sequences.

### Diversity analyses

The diversity of the strains was determined using multilocus typing MLST on the assembled genomes. For phylogeny, two approaches were used: (1) a gene by gene cgMLST phylogenetic analysis was performed in enterobase (https://enterobase.warwick.ac.uk/) based on allelic distances; and (2) single nucleotide polymorphisms-based phylogenetic analysis (SNPs) were also performed to assess the genetic relatedness of isolates across niches. For the SNPs analysis, we followed previously described procedures^21^ where variants were called with Snippy v4.6.0 (https://github.com/tseemann/snippy) under the following parameters: mapping quality of 60, a minimum base quality of 13, a minimum read coverage of 4, and a 75% concordance at a locus. An alignment of core genome variants was produced with *snippy-core* for phylogeny inference. Putative recombinogenic regions were detected and masked with Gubbins version v2.4.1^27^. A maximum likelihood (ML) phylogenetic tree was built, with RAxML version/8.2.12, under the GTR model with 1000 bootstraps^28^. The final tree was rooted on the reference *E. coli* K12 substr MG1655 genome and visualized with iTOL v5^29^. The pairwise SNP distance was generated with *snp-dists* v 0.8.2 (https://github.com/tseemann/snp-dists) and the data is provided in Table S2. Given the study period lasting from August to February and knowing the general heterogeneity of the *E. coli* genomes, strains with less than 50 SNP difference were considered genetically related.

### Resistome and mobilome analyses

A systematic resistome analysis was performed using combined database from Resfinder v4.1^30^ and AMRfinderplus v3.10.24^31^. Phenotypic and genotypic resistome data was subjected to a pooled analysis, where comparisons of relative frequencies for resistance and the abundance of resistance gene by antimicrobial classes was performed according to place of isolation.

Analysis of the mobile genetic elements hosting the main ESBL gene was performed using annotated genomes with comparison and visualization of the genetic context around the main ESBL gene using clinker^32^ and BLAST atlas.

All genomes and assembly fragments were annotated using Bakta v1.5.1^33^. With the complete assemblies, the genetic contexts of ESBL resistance genes were observed in Artemis, and genomic resistance islands were identified using the IslandViewer 4 tool^34^. Insertion sequence (IS) elements were identified using the BLAST tool on the ISfinder database (https://www-is.biotoul.fr/blast.php). Using GView Server (https://server.gview.ca/), we mapped the reconstructed plasmids against their closest hits from NCBI to determine representativeness and some genetic context visualizations were performed with CLC Main Genomics workbench (Qiagen).

### Plotting and statistical analysis

Data were plotted in R v4.2.1 using ggplot2. For resistome analysis similarity analysis was performed using the Jaccard similarity index (JI) (between 0 and 1, where 1 is identical), and the resulting JIs were used to generate a hierarchical clustering heatmap in Rv 4.2.1.

### Ethics approval

Ethical approval for the study was obtained from Makerere University School of Public Health Higher Degrees Research and Ethics Committee (HDREC #760) and Uganda National Council for Science and Technology (UNCST) (#HS649ES). The study also obtained administrative approval from Uganda Ministry of Health (#ADM.185/130/01), while written informed consent was obtained from each eligible study participant with assurance for confidentiality of information. All methods in this study were performed in accordance with the relevant guidelines and regulations.

## Supplementary Information

Supplementary Figure 1.

Supplementary Figure 2.

Supplementary Legends.

Supplementary Tables.

## Data Availability

All data described in this study are available in the supplementary excel workbook. The sequenced genomes both on Illumina and Nanopore are deposited to the European Nucleotide Archive under the project accession number PRJEB59977 (data available at: https://www.ebi.ac.uk/ena/browser/view/PRJEB59977).
