# Supplementary figures and images for: Whole strains vs MGEs in short and longterm transmission of ESBL genes between healthcare and community settings in Uganda
Source: Sci Rep. 2023 Jun 23;13:10229. doi: 10.1038/s41598-023-35879-x (PMC10290109; doi:10.1038/s41598-023-35879-x)

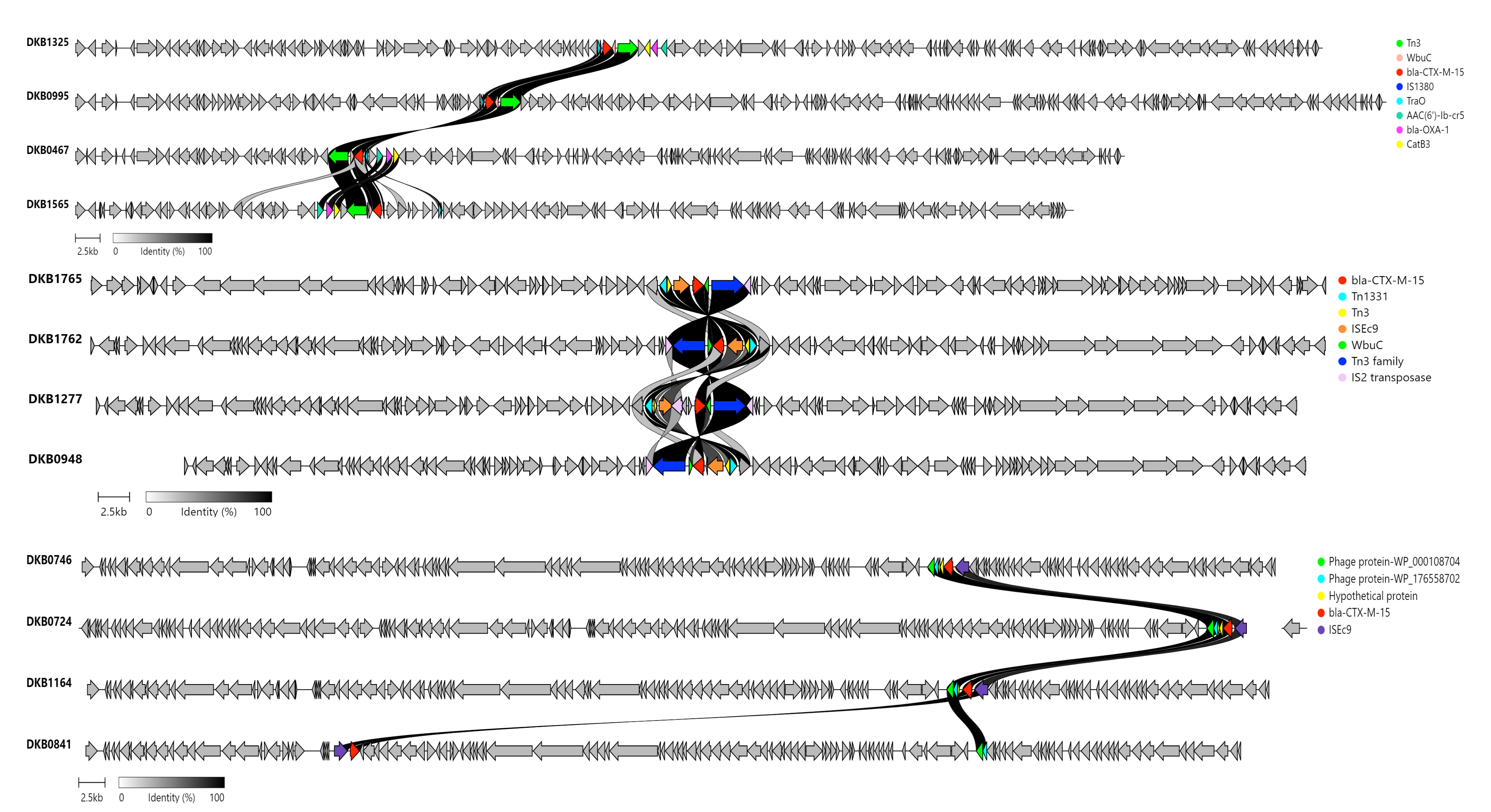

Supplement: Supplementary file 1 — Supplementary Figure 1. [file 41598_2023_35879_MOESM1_ESM.png]

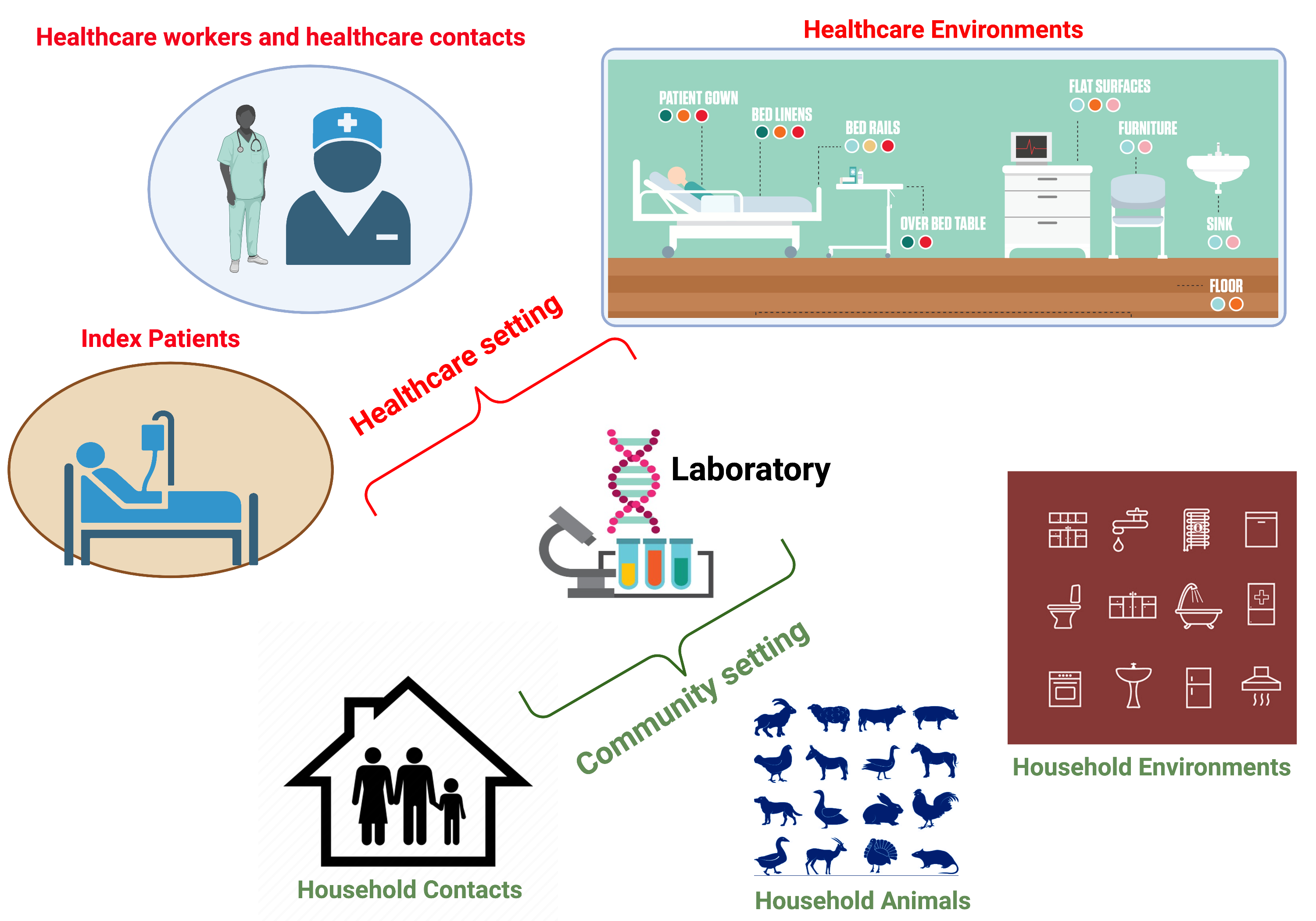

Supplement: Supplementary file 2 — Supplementary Figure 2. [file 41598_2023_35879_MOESM2_ESM.png]
