# Supplementary material for: Whole strains vs MGEs in short and longterm transmission of ESBL genes between healthcare and community settings in Uganda
Source: Sci Rep. 2023 Jun 23;13:10229. doi: 10.1038/s41598-023-35879-x (PMC10290109; doi:10.1038/s41598-023-35879-x)
Supplement: Supplementary file 3 — Supplementary Legends. [file 41598_2023_35879_MOESM3_ESM.docx]

**Legends for Supplementary Materials**

**Figure S1.** Genetic contexts around *bla-*_CTX-M-15_ in each cluster of plasmid in strains isolated from healthcare and community settings. The legend to the right side for each plasmid cluster shows a colored annotation of coding sequences around *bla-*_CTX-M-15_ in that category of plasmid.

**Figure S2.** Diagram of the sampling frame showing the different sampled niches. Chart made in premium Biorender.

**Table S1:** AMR Genes detected per isolate

**Table S2:** ST detected in sequenced samples

**Table S3**: Pairwise SNP Matrix

**Table S4:** Characteristics of plasmids reconstructed from hydrid assemblies

**Table S5:** Overall samples metadata and AST data

**Table S6:** ESBL producing isolates included in the study
